# Intestinal Tuberculosis With Recurrent Gluteal Abscesses and Perianal Fistulas: A Case Report and Literature Review

**DOI:** 10.7759/cureus.76456

**Published:** 2024-12-27

**Authors:** Igor Takakazu Ribas Asato, Nayane Souza Novaes Asato, Fernanda Amariz Yamamoto, Ana Caroline Ferreira Siqueira, Anderson M Pereira da Silva

**Affiliations:** 1 Family and Community Medicine, Secretaria Municipal de Saúde de Campo Grande, Campo Grande, BRA; 2 Medicine, Universidad Internacional Tres Fronteras, Campo Grande, BRA; 3 Pharmacology, Universidade Federal do Vale do São Francisco, Petrolina, BRA

**Keywords:** extra-pulmonary tuberculosis (eptb), intestinal tuberculosis, low lying perianal fistulas, perianal fistulas, recurrent abscess

## Abstract

This case is relevant for describing a rare presentation of intestinal tuberculosis with perianal manifestations, complicated by abscesses and recurrent fistulas. The clinical manifestations mimicked Crohn's disease and other inflammatory conditions, making the diagnosis challenging and requiring a differentiated and meticulous diagnostic process.

A 45-year-old male patient presented with a chronic abscess in the left buttock lasting for two years, characterized by spontaneous purulent drainage and multiple recurrences despite surgical and clinical treatments. Imaging findings revealed associated fistulous lesions, initially interpreted as possible inflammatory complications.

The definitive diagnosis was confirmed as intestinal tuberculosis with perianal involvement through colonoscopy and biopsy, which revealed caseating granulomas. Treatment included a standard antituberculous regimen (rifampin, isoniazid, pyrazinamide, and ethambutol), along with antibiotics and antifungal agents for secondary infections. Adherence to treatment was satisfactory, with progressive improvement in the fistulas. This case highlights the importance of a comprehensive differential diagnosis in extrapulmonary tuberculosis, especially with unusual manifestations. Multidisciplinary management was essential for therapeutic success and the prevention of recurrences.

## Introduction

Tuberculosis is an infectious disease caused by *Mycobacterium tuberculosis*, which, while predominantly affecting the lungs, can also involve other organs, such as the gastrointestinal tract [1,2]. Intestinal tuberculosis is a rare form of the disease, posing a diagnostic challenge due to its similarity to other conditions, such as Crohn's disease and fungal infections, such as abdominal pain, chronic diarrhea, weight loss, fever, and inflammatory intestinal lesions that can lead to ulcerations and strictures [3,4]. In this report, we discuss a case of intestinal tuberculosis that presented with multiple perianal fistulas and recurrent abscesses.

The identification of intestinal tuberculosis can be hindered by its nonspecific clinical presentation, which includes abdominal pain, weight loss, fever, and symptoms related to abscess and fistula formation [4,5]. In some cases, as in the one reported here, the patient may develop complications mimicking inflammatory bowel diseases, which can lead to initial misdiagnosed approaches. Previous reports emphasize that diagnostic confirmation often relies on histopathological findings, such as the presence of caseating granulomas, and the exclusion of conditions like neoplasms and actinomycosis. These approaches are critical to avoid inappropriate treatments [6,7].

In this context, the present case report describes the clinical evolution, diagnostic methods, and treatment of a patient with intestinal tuberculosis complicated by perianal fistulas and recurrent abscesses.

## Case presentation

**Figure 1:**
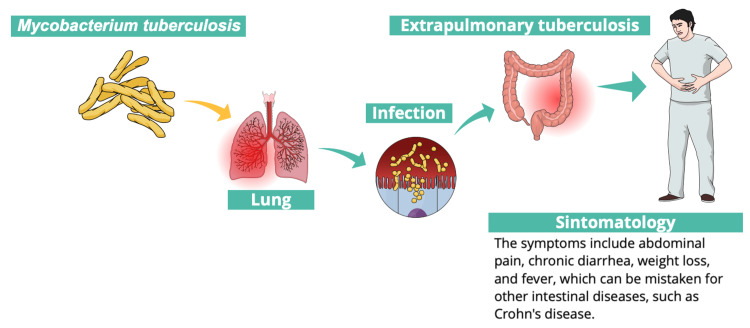
Pathophysiological progression of intestinal tuberculosis Figure created by authors with mindthegraph.com

The patient, a 45-year-old male, works as a gas station attendant and is married. He had no family history of tuberculosis. Among the main symptoms, he presented with a recurrent abscess in the left buttock, characterized by spontaneous purulent discharge, local pain, and frequent relapses. The patient had a history of four months of incarceration in 2021, which exposed him to living and working conditions that may have increased his risk of tuberculosis infection. Figure 1 provides a detailed representation of the pathogenesis associated with the intestinal tuberculosis case.

Previous interventions included surgical drainage of the abscesses, empirical antibiotic therapy, and colonoscopies to investigate a possible inflammatory bowel disease. Physical examination revealed an ulcerated lesion in the gluteal region with signs of an active fistula and purulent discharge. No palpable peripheral lymphadenopathy was identified. However, the proctological exam revealed multiple perianal fistulas.

The patient underwent various diagnostic methods, including colonoscopy with intestinal biopsy, musculoskeletal ultrasound, and laboratory tests such as complete blood count and secretion culture. A significant diagnostic challenge was the clinical similarity to Crohn’s disease, necessitating detailed histopathological evaluation and exclusion of other differential diagnoses such as actinomycosis and neoplasms. Complementary examinations confirmed the diagnosis of intestinal tuberculosis based on histopathological findings of caseating granulomas.

The patient’s prognosis was guarded, requiring continuous follow-up to monitor treatment response and prevent potential complications, such as the emergence of new fistulas or abscesses. Therapeutic management involved a pharmacological approach with the administration of rifampin, isoniazid, pyrazinamide, and ethambutol, as well as surgical interventions for fistula drainage and local wound care. Antibiotics and antifungal agents were also administered to control secondary infections.

The treatment regimen included rifampin 150 mg, isoniazid 75 mg, pyrazinamide 400 mg, and ethambutol 275 mg, with a planned duration of six months. Additional antibiotics and antifungal agents were introduced due to secondary infections that did not respond adequately to the initial treatment. Clinical follow-up revealed significant improvement in the healing of perianal lesions and control of secondary infections, with the patient reporting symptomatic relief and reduced purulent discharge.

Follow-up tests, including complete blood counts and liver function tests, showed no significant abnormalities. The patient demonstrated good adherence to treatment and tolerance to the prescribed medications, and no relevant adverse events were observed during the course of therapy.

## Discussion

Intestinal tuberculosis, while uncommon, constitutes a clinically significant extrapulmonary manifestation of *Mycobacterium tuberculosis *infection. Its nonspecific clinical presentation often results in delayed diagnosis and treatment, complicating disease management. In the present case, recurrent perianal abscesses and fistulas were the predominant manifestations, an atypical presentation rarely documented in the literature. This form of tuberculosis is frequently misinterpreted as other conditions, such as Crohn’s disease, due to overlapping clinical and histopathological characteristics, which underscores the considerable diagnostic challenges associated with this condition [5,8-10].

A key strength of this report was the correct identification of intestinal tuberculosis following the exclusion of complex differential diagnoses, such as inflammatory bowel diseases and neoplasms. Careful exclusion of other conditions was critical as inappropriate treatments could have exacerbated the patient’s complications. The finding of caseating granulomas in the intestinal biopsy was pivotal for the diagnosis. Additionally, differences in transmission pathways between gastrointestinal and pulmonary tuberculosis warrant attention. Pulmonary tuberculosis is primarily transmitted via airborne droplets, whereas intestinal tuberculosis typically arises from the ingestion of food or water contaminated with *Mycobacterium tuberculosis* or the swallowing of infected pulmonary secretions in patients with active tuberculosis. Risk factors specific to intestinal tuberculosis include immunosuppressive conditions, such as HIV infection, chronic malnutrition, and prolonged exposure to high-risk environments, such as prisons or areas with poor sanitation.

These factors differ substantially from pulmonary tuberculosis, which relies more directly on aerosolized particle exposure. From a therapeutic perspective, the positive response to the antituberculous regimen, comprising rifampin, isoniazid, pyrazinamide, and ethambutol, was crucial for the patient’s favorable outcome. The concomitant use of antibiotics and antifungal agents to manage secondary infections was an important component of successful treatment. The progressive healing of fistulas and the symptomatic relief reported by the patient indicate the efficacy of the chosen therapeutic approach.

However, a significant limitation was the initial delay in reaching the correct diagnosis, which postponed the start of antituberculous therapy. This delay likely contributed to the recurrence of abscesses and the progression of fistulas, further complicating the patient’s clinical course.

## Conclusions

This case report provides valuable lessons for clinical practice, especially in regions endemic for tuberculosis, highlighting the importance of considering extrapulmonary tuberculosis in the differential diagnosis of chronic abscesses and perianal fistulas. Prompt exclusion of differential diagnoses and the implementation of appropriate therapies can significantly improve the prognosis and reduce complications associated with this rare condition.
